# Pancreatoblastoma in a paediatric patient: anatomo-pathological aspects of a case with multiple hepatic metastases

**DOI:** 10.3332/ecancer.2018.861

**Published:** 2018-08-22

**Authors:** Gabriel Cao, Julián Mendez, Daniel Navacchia

**Affiliations:** 1 Anatomical Pathology Division, Pedro De Elizalde Children’s Hospital, Avenida Montes de Oca 40 (C1270AAN), Buenos Aires, Argentina; 2 National Council of Scientific and Technical Research (CONICET), Godoy Cruz 2290 (C1425FQB), Buenos Aires, Argentina

**Keywords:** pediatric pancreatoblastoma, pathology, treatment

## Abstract

Pancreatoblastoma is a rare paediatric malignant neoplasm. The treatment of choice is complete surgical resection. However, it is often unresectable due to its large size, local infiltration or distant metastasis. Since the condition is rare, there is currently no standard treatment regimen. We outline the case of a 4-year-old child who presented with abdominal pain and distention, together with an enlarged liver and elevated serum α-fetoprotein levels. Imaging studies showed the presence of an abnormal pancreatic tumour and multiple nodular lesions in the liver, the biopsies from which led to a diagnosis of pancreatoblastoma. In this case, the patient received cycles of neoadjuvant chemotherapy, combining cisplatin and doxorubicin. The patient subsequently underwent scheduled surgery in which the primary pancreatic lesion was resected, obtaining a circumscribed and nodular specimen measuring 7 × 6 cm and weighing 150 g. Given the extent of the metastasis, the child is currently awaiting a liver transplant.

## Introduction

Pancreatoblastoma is the term given to rare malignant neoplasms [1] that mimic foetal pancreatic development at 7 weeks of gestation [2]. It tends to affect young, predominantly male, children, presenting clinically as an abdominal mass, abdominal pain or obstructive jaundice [3]. The rarity of the condition and the lack of specificity in its symptoms make pancreatoblastoma a real diagnostic challenge for oncologists and surgeons, especially since standardised guidelines are not available for this purpose [1, 4]. Since it is a biologically aggressive neoplasm, it may be unresectable at the time of diagnosis, instead of requiring neoadjuvant chemotherapy to shrink the tumour. In localised cases, complete resection of the lesion is required. However, management is problematic when the neoplasia spreads outside of the pancreas or causes distant metastasis since, again, there is no standardised treatment regimen available [4]. To contribute to this field, we outline the case of a 4-year-old child with a pancreatoblastoma and multiple liver metastases at the time of diagnosis.

## Clinical case

A 4-year-old male Caucasian patient with no prior family history of neoplasms presented in fair general condition with weight loss and loss of appetite, which, according to his parents, had developed over 2 months. Clinical evaluation showed evidence of abdominal pain and distention in addition to hepatomegaly. A full work-up was requested, significant findings were anaemia, very high levels of α-fetoprotein (352,050 ng/mL, normal range: <20 ng/mL), low levels of chorionic gonadotropin subunit beta (1.9 mIU/mL) and elevated levels of lactate dehydrogenase (1,615 IU/L). Tests on the levels of catecholamines in the blood and urine were negative. An abdominal ultrasound was performed, which showed irregular hepatomegaly with a heterogeneous echotexture due to the presence of multiple, randomly distributed echogenic nodules, suggestive of metastasis. The pancreas could not be evaluated. This was followed by an abdominal and pelvic CT scan with and without contrast dye: this showed a heterogeneous tumour in the body and tail of the pancreas that had defined borders, hypodense areas inside (suggestive of necrosis) and an apparent pseudocapsule. This measured: anteroposterior diameter 75 × transverse diameter 57 × longitude 101 mm, displacing the left kidney and the splenic artery in the head and rear directions, the left ureter in the dorsal direction and the bowel in the caudal direction. Enlarged heterogeneous liver, due to the presence of multiple hypodense nodules. The full body bone scan pathology did not show focal uptake. The adrenal glands showed no significant changes.

The patient’s condition deteriorated: he presented an acute abdomen requiring surgery, for which an exploratory laparotomy was performed. This revealed a hepatic nodular lesion in the left lobe, with surface erosion and bleeding, from which biopsies were taken. The histopathological study with Haematoxylin–Eosin (H & E) staining showed limited liver trabeculae, infiltrated by a poorly differentiated malignant neoplastic proliferation consisting of medium-sized round or polygonal cells with large cytoplasma, with areas of necrosis and haemorrhage. Immunohistochemistry procedures were run, revealing neoplastic cells: positive for vimentin, Cytokeratin (AE1–AE3), β-catenin, Ki-67 (in 80% of the neoplastic nuclei) and, focally, carcinoembryonic antigen. In contrast, the tests were negative for neuron-specific enolase, Chromogranin and Hep Par-1. The earlier findings, together with the patient’s clinical context, suggested liver metastasis due to pancreatoblastoma.

The patient went into intensive care and, upon returning to the ward, started a course of chemotherapy, using cisplatin 56 mg/m^2^ and doxorubicin 21 mg/m^2^.

Following neoadjuvant treatment, the patient underwent schedule surgery for excision of a pancreatic tumour. The surgical approach was through the lesser sac, locating it in the tail and body of the pancreas. We proceeded to the lower margin, releasing the relevant vessels, to the posterior for unaffected pancreatic tissue and, finally, to the upper margin respecting the splenic vessels. A circumscribed, nodular lesion of 7 × 6 cm and weighing 150 g was obtained in the aforementioned procedure (pancreatectomy of the body and tail). The site of incision had a yellowish surface with solid areas and a friable central section. Histological sections showed the presence of a pancreatoblastoma, with large areas of necrosis and two residual peripheral nodules. Additional immunohistochemical techniques demonstrated the membrane positivity for E-cadherin and vascular endothelial growth factor (VEGF).

At present, the patient is receiving medical follow-up and is in a liver transplant programme.

## Discussion

Frable *et al* [5] were the first to give histological and ultrastructural characterisation to pancreatoblastoma, while Horie *et al* [6] also made contributions regarding its possible morphogenesis. In this regard, it was suggested pancreatoblastoma originates in the ventral pancreatic bud as a result of abnormal embryological development [6, 7], which would explain why it is typically found at the head of the pancreas. However, it can sometimes be found in the tail of the pancreas [8].

Pancreatoblastoma accounts for 0.2% of all neoplasms in the pancreas, being most frequent among children (mean age of 5 years) [9, 10]. In a cohort study, it accounted for 16% of all pancreatic primary malignant neoplasms among children, with an average age at presentation of 5.5 years [11]. The aforementioned study also notes that it can occur within the context of a hereditary syndrome such as Beckwith–Wiedemann or Familial adenomatous polyposis syndrome, suggesting a pathogenesis related to alterations in chromosomes 11 and 5, respectively [3, 11]. It originates in the epithelial cells of the exocrine sections of the pancreas, as with adenocarcinoma, adenosquamous carcinoma, acidic cell carcinoma and solid pseudopapillary tumour [12]. Its slow growth means that it often occurs as a large abdominal mass, which usually hinders identification of the organ in which it originates [13]. Furthermore, it also tends to present with elevated serum levels of α-fetoprotein [14] due to the common embryologic development of the pancreas and liver, both of which originate in the foregut. In these circumstances, it can be used to indicate how well a patient is responding to treatment and to monitor progress [7, 15].

Macroscopically, the lesion is usually enclosed, lobed and solid with a heterogeneous, necrotic, haemorrhagic cut surface and a mean diameter that can reach up to 11 cm [16, 17]. Microscopically, the neoplasia is characterised by being richly cellular, cytologically uniform and organised in nests and islets, with a tendency to form acinar structures [2, 3, 6, 15]. An important feature of diagnostic value is the formation of whorled nests of scaly spindle cells (squamoid corpuscules), which exhibit occasional keratinisation, contributing to the differential diagnosis of acinar cell carcinoma. Tumour stroma is relatively common, especially among children, which may eventually show heterologous elements such as bone or cartilage.

Immunohistochemical techniques show diffuse expression of Cytokeratin membrane (AE1–AE3) and epithelial membrane antigen, while in areas of acinar differentiation one tends to find typical pancreatic exocrine enzymes such as trypsin, chymotrypsin and lipase, the latter to a lesser extent [17]. In addition, in solid sectors without clear acinar differentiation, pancreatoblastoma can display immunoreactivity to chromogranin, synaptophysin and neuronal specific enolase, which hinders the differential diagnosis especially with small biopsies. In this regard, some authors consider that aberrant nuclear expression of β-catenin and the loss of expression of E-cadherin membrane are characteristics of the solid pseudopapillary tumour of the pancreas, another factor that must be considered in the differential diagnosis [18]. VEGF is a pro-angiogenic growth factor involved not only in normal pancreatic development but also in tumour growth and metastasis [19]. In the case study presented, the immunohistochemical expression was intense and diffuse in the primary pancreatic lesion, coinciding with the onset of metastasis.

In the study conducted by Bien *et al* [1], complete resection is the treatment of choice, whether it be at the time of diagnosis or following chemotherapy treatment (5-year survival rate: 30%–50%). In this regard, they do not consider there to be a correlation between tumour size and the ability to perform a complete resection nor between the tumour’s size and its progression. However, in cases where the tumour is technically unresectable, prior chemotherapy treatment is acceptable, with optimal response to the use of drugs such as cyclophosphamide, etoposide, cisplatin or doxorubicin [1, 12, 20]. In these cases, radiotherapy could also play a role, although its use is limited given the morbidity that may result, especially considering that patients with pancreatoblastoma are usually young children [1]. The study also raises the option of liver transplantation in those cases with massive metastasis on the liver [4].

Finally, the work done by Dhebri *et al* [21] outlines certain factors influencing the prognosis of patients with pancreatoblastoma. Univariate analysis showed that the presence of synchronous or metachronous metastasis, unresectability of the lesion at the time of diagnosis and being over 16 years old worsen the prognosis. On the other hand, multivariate analysis found that complete surgical resection and the development of post-operative metastasis independently influence the long-term survival. The same is not true of local recurrences.

## Conclusion

Pancreatoblastoma is a rare malignant neoplasm, which usually presents as a slow-growing abdominal mass and with high serum levels of α-fetoprotein. This should be always considered in a young child. It represents a real diagnostic challenge for oncologists, surgeons and pathologists. For this reason, it is necessary to gather as much clinical and additional information as possible when a surgical specimen is taken. The treatment of choice is a complete resection of the primary neoplasia, although this can also include neoadjuvant chemotherapy and, in cases with massive metastasis, liver transplantation. The case study illustrates the characteristics of the disease and the strategies currently available to approach a patient’s treatment.

## Conflicts of interest

The authors have no conflicts of interests to declare.

## Authors’ contributions

All authors participated in the bibliographical research and preparation of the manuscript. All authors approved the final version of the same.

## Funding

The authors declare that the work submitted has not received specific financial support from the public sector, commercial bodies or non-profit entities.

## Figures and Tables

**Figure 1.**
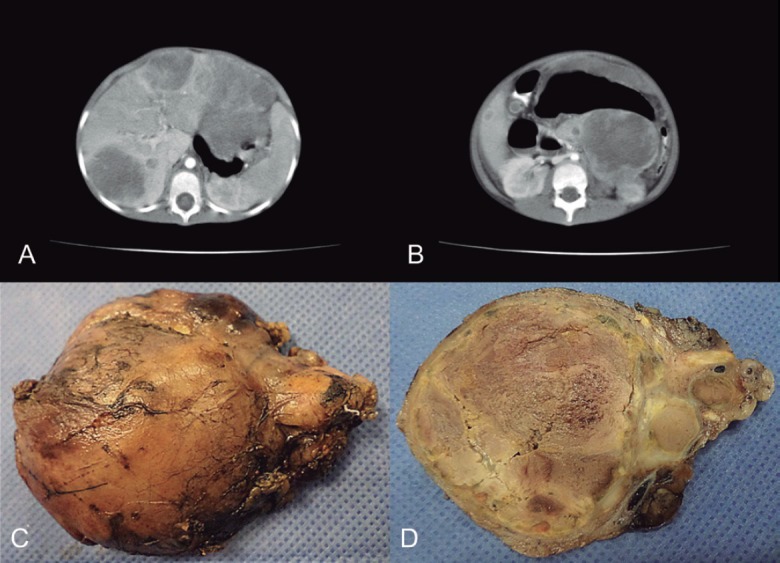
(a) CT scan that shows enlarged, heterogeneous liver due to the presence of multiple hypodense nodules. (b) In the body and tail of pancreas, there was a heterogeneous tumour with defined boundaries and hypodense areas inside, suggestive of necrosis, together with an apparent pseudocapsula. (c) Macroscopic appearance of the pancreatic lesion measuring 7 × 6 cm and weighing 150 g, circumscribed, nodular with the congestive outer surface. (d) Yellowish cut surface with solid areas and friable central sector, necrotic.

**Figure 2.**
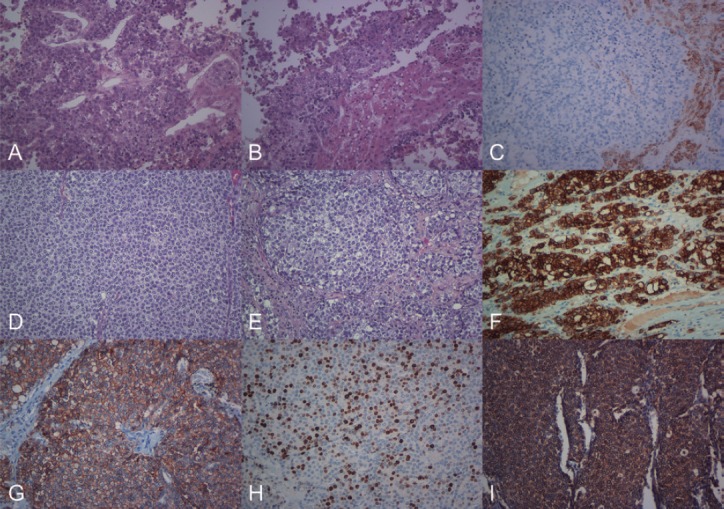
(a) Histological image showing the biopsy of the liver nodules, at the start of the diagnostic process (H & E 200×). (b) In relation to the neoplastic proliferation, residual hepatic trabeculae were observed (H & E, 200×). (c) The immunohistochemical expression for Hep-Par1 was positive only in the liver parenchyma (immunohistochemistry, 200×). (d) Histological image showing the surgical resection of the pancreatic tumour, the characteristics of which were similar to those observed in the previous liver biopsy (H & E, 200×). (e) Neoplastic cells showed moderate anicocariosis, visible nucleoli and extensive, occasionally vacuolated, cytoplasm (H & E, 200×), which were positive for Cytokeratin (f). E-cadherin (g), Ki67 in 80% of the proliferation (h) and VEGF (i) (immunohistochemistry 200×).
